# Survival, growth and stress response of juvenile tidewater goby, *Eucyclogobius newberryi*, to interspecific competition for food

**DOI:** 10.1093/conphys/cow013

**Published:** 2016-04-22

**Authors:** Daniel A Chase, Erin E Flynn, Anne E Todgham

**Affiliations:** Department of Animal Science, University of California Davis, One Shields Avenue, Davis, CA 95616, USA

**Keywords:** Estuarine fishes, food availability, generalized stress response, interspecific competition, tidewater goby

## Abstract

Reintroduction of endangered fishes to historic habitat has been used as a recovery tool; however, these fish may face competition from other fishes that established in their native habitat since extirpation. This study investigated the physiological response of tidewater goby, *Eucyclogobius newberryi*, an endangered California fish, when competing for food with threespine stickleback, *Gasterosteus aculeatus*, a native species, and rainwater killifish, *Lucania parva*, a non-native species. Survival, growth and physiological indicators of stress (i.e. cortisol, glucose and lactate concentrations) were assessed for juvenile fish held for 28 days in two food-limited conditions. When fed a 75% ration, survival of *E. newberryi* was significantly lower when held with *G. aculeatus*. In all fish assemblages, weight and relative condition decreased then stabilized over the 28 day experiment, while length remained unchanged. Whole-body cortisol in *E. newberryi* was not affected by fish assemblage; however, glucose and lactate concentrations were significantly higher with conspecifics than with other fish assemblages. When fed a 50% ration, survival of *E. newberryi* decreased during the second half of the experiment, while weight and relative condition decreased and length remained unchanged in all three fish assemblages. Cortisol concentrations were significantly higher for all fish assemblages compared with concentrations at the start of the experiment, whereas glucose and lactate concentrations were depressed relative to concentrations at the start of the experiment, with the magnitude of decrease dependent on the species assemblage. Our findings indicate that *E. newberryi* exhibited reduced growth and an elevated generalized stress response during low food availability. In response to reduced food availability, competition with *G. aculeatus* had the greatest physiological effect on *E. newberryi*, with minimal effects from the non-native *L. parva*. This study presents the first reported cortisol, glucose and lactate concentrations in response to chronic stress for *E. newberryi*.

## Teaser

Understanding the potential effect of competition with native and non-native fishes is essential for reintroduction efforts of extirpated fishes. We investigated the physiological effects of competition for food in the California endangered tidewater goby. Greater competition existed with the native species and the goby stress response varied with food level.

## Introduction

Competition between organisms for limited food resources can force individuals to be excluded or displaced from otherwise suitable habitat (Mills *et al*., 2004; Ward *et al*., 2006; Seiler and Keeley, 2009; Mac Nally *et al*., 2010; Britton *et al*., 2011). Alternatively, habitat that might otherwise support multiple species that share similar trophic niches may become limited in capacity during periods of low food availability (Mac Nally *et al*., 2010). This effect has been shown for both intra- and interspecific competition among fishes, and the intensity of competition effects can vary based on life stage and season (Osenberg *et al*., 1992; Mills *et al*., 2004; Ward *et al*., 2006; Blanchet *et al*., 2007). The stronger competitive species can monopolize limited food resources, causing the less dominant species to become displaced or excluded from the habitat (McDowall, 2003). When an introduced species occupies the more dominant role, native fish can suffer more during low-food conditions than high-food conditions, as the resources can drive species interactions and shape community composition (Gunckel *et al*., 2002). The introduction of non-native species has altered species assemblages and food web compositions in the aquatic environment and challenged the conservation of imperilled species (Marchetti *et al*., 2001). The dynamics of competition for food between native and non-native species when food is limited is an important consideration when trying to understand species interactions in the wild.

On the individual level, competition for limited resources presents physiological challenges that can affect energy allocation to key biological processes, such as growth and development (Kassahn *et al*., 2009). Competition can trigger the activation of a fish's generalized stress response to aid the individual in acquiring the energy necessary for ‘fight or flight’ through the mobilization of glucose (Barton, 2002; Kassahn *et al*., 2009). Repeated or chronic stress can place a high energy demand on an individual (Barton, 2002; Cook *et al*., 2012), and therefore, when food is limited, an organism may not have sufficient energy reserves to maintain homeostasis (DiBattista *et al*., 2006). For juvenile fish, food availability and chronic exposure to competition can have a significant impact on growth during a crucial period of development and can ultimately prolong the timing of sexual maturation (Wendelaar Bonga, 1997; Barton, 2002; McGhee and Travis, 2011). For annual and short-lived species, the delayed growth, even over short periods of time, can result in lower fecundity and impaired mate selection (Kassahn *et al*., 2009; McGhee and Travis, 2011) and can therefore have repercussions at the population level.

One such annual species that occurs in highly variable habitats is the tidewater goby, *Eucyclogobius newberryi*, an endangered fish species endemic to California coastal lagoons and estuaries. Given that the species has a short lifespan in the wild and that estuarine habitat can be dynamic and variable, quick growth and high reproductive output are important to the local abundance of the species and are factors in maintaining the resilience of a population (Lafferty *et al*., 1999a,b; US Fish and Wildlife Service, 2005). After a brief pelagic larval stage, the *E. newberryi* juvenile stage may only last a few weeks, when the fish is 9.8–24.8 mm standard length (SL), and ends when sexual maturity is achieved (potentially starting at 25 mm SL for males and 27 mm SL for females; Swift *et al*., 1989; Swenson, 1999; Spies and Steele, 2016). Fecundity is positively associated with size in *E. newberryi*, and for a species that typically lives only a year in the wild, a delay or impairment in growth during the juvenile stage that results in smaller females or delayed sexual maturation could compromise the quantity of young produced and the number of times that reproduction can occur (Swift *et al*., 1989; Swenson, 1993, 1999; McGourty *et al*., 2008). Understanding the physiological effects of competition for food on *E. newberryi* is important when considering an effort to re-establish a population in an area the species historically occupied, such as the San Francisco Bay Estuary. The alteration of coastal estuaries and the introduction of non-native species have contributed to the extirpation of *E. newberryi* in portions of the species' historic range (US Fish and Wildlife Service, 2005). Recovery efforts to restore and improve habitat for the species have been conducted; however, no studies have been performed to establish whether biotic interactions between *E. newberryi* and introduced non-native fishes might compromise recovery efforts owing to competition for food.

Several factors can influence the success of reintroduction of an endangered species, including habitat condition, proximity to other populations, life-history requirements and other species currently found in the habitat (US Fish and Wildlife Service, 2005; Anderson *et al*., 2014). While predatory species can threaten successful reintroduction, more subtle effects, such as competition for limited food resources between juvenile species, could also factor into the success of a targeted reintroduction. Previous research investigating the social interaction of *E. newberryi* in ample food conditions found no impact of species assemblage on survival, growth or stress hormone concentrations (i.e. cortisol); however, behavioural observations during feeding provided evidence for competition if rations of food were reduced (Chase and Todgham, 2016). The present experiment was designed to examine the role of interspecific competition for food on the physiological condition of *E. newberryi*. By manipulating the feeding ration at two levels (75 and 50% ration), survival, growth and primary and secondary physiological indicators of stress (i.e. whole-body cortisol, glucose and lactate) of *E. newberryi* were examined in response to competition with the non-native rainwater killifish, *Lucania parva*, and the native threespine stickleback, *Gasterosteus aculeatus. Eucyclogobius newberryi* and *G. aculeatus* co-occur in the wild; however, *L. parva* and *E. newberryi* do not. *Lucania parva* has become established in habitat historically occupied by *E. newberryi* in the San Francisco Bay Estuary, and *L. parva* represents a novel species interaction for *E. newberryi* (US Fish and Wildlife Service, 2005)*.* By studying these species paired with *E. newberryi* in two feeding regimens, in this experiment we sought to investigate the potential role of competition with a non-native species on *E. newberryi*, with the objective of better informing evaluation measures for reintroduction of this endangered species.

## Materials and methods

### Field collection and animal holding

Juvenile *E. newberryi* were collected using seine nets on 9 July 2013 and 15 August 2013 from Salmon Creek Lagoon, in Sonoma County, CA, USA (38°21′11.42″N, 123° 3′57.02″W). Water temperature, salinity and dissolved oxygen at Salmon Creek Lagoon during collection were 19.3°C, 0.2 ppt and 7.99 mg O_2_ l^−1^, respectively, on 9 July and 18.7°C, 0.3 ppt and 8.03 mg O_2_ l^−1^, respectively, on 15 August. Juvenile *G. aculeatus* and *L. parva* were collected using seine nets on 15 July 2013 and 19 August 2013 from lower Coyote Creek watershed in Marin County, CA, USA (37°52'34.09″N, 122°31'35.11″W). Water temperature, salinity and dissolved oxygen within lower Coyote Creek during collection were 18.9°C, 29.3 ppt and 1.79 mg O_2_ l^−1^, respectively, on 15 July and 16.9°C, 29.7 ppt and 2.69 mg O_2_ l^−1^, respectively, on 19 August. Juvenile fish of similar size to *E. newberryi* were size selected in the field and transported using aerated coolers to the Romberg Tiburon Center for Environmental Studies, San Francisco State University in Tiburon, CA, USA. Fish were collected over two different periods to accommodate the two different feeding trials (i.e. July for the 50% ration and August for the 75% ration; details below). All fish were grouped separately by species and were held in indoor, recirculating aquaria under natural photoperiod until experimentation. Juvenile *E. newberryi* fish used in the experiments ranged in size from 18.0 to 25.0 mm SL, with a mean size of 21.6 ± 1.5 mm SL.

Salinity in all aquaria was gradually adjusted through partial water changes at a rate of 2 ppt day^−1^ from the salinity measured at the site of collection for each group of fishes to a salinity of 25 ppt. A salinity of 25 ppt was selected to simulate mean summer salinity conditions within San Francisco Bay Estuary habitat historically occupied by *E. newberryi*. Using US Geological Survey Corte Madera Creek water-quality station (gauge 11460090), mean salinity for 1 June–31 October over a 3 year period was calculated to determine the experiment salinity level. The mean salinity level of 25 ppt falls within the range of occupied habitat and physiological tolerance for *E. newberryi* (Chamberlain, 2006), *G. aculeatus* (Moyle, 2002) and *L. parva* (Fuller *et al*., 2007). *Eucyclogobius newberryi* were held in aquaria at 5 ppt for 3–4 days before beginning incremental salinity increases over 10–11 days (from 5 to 25 ppt), whereas salinity adjustments in aquaria of *G. aculeatus* and *L. parva* took 2–3 days (from 29 to 25 ppt). Incremental salinity increases were achieved through partial water changes. Once a salinity of 25 ppt was reached in all aquaria, *E. newberryi* were held in these conditions for 1–3 days, whereas *G. aculeatus* and *L. parva* were held in these conditions for 9 days, before the start of the feeding trials. Aquaria temperatures were maintained at 19 ± 3°C and dissolved oxygen levels at 7.9 ± 0.9 mg O_2_ l^−1^ for the duration of the laboratory acclimation period.

Authorization for fish collection and study design approval was granted under US Fish and Wildlife Service Recovery Permit number TE237061-1, California Department of Fish and Wildlife Scientific Collectors Permit number SC-8472 and a State of California Department of Parks and Recreation Collection Permit. Experimental procedures, handling and care were reviewed and approved by the San Francisco State Institutional Animal Care and Use Committee (IACUC Protocol #A12-04).

### Experimental design

Juvenile *E. newberryi* growth, survival and physiological indicators of stress (i.e. whole-body cortisol, glucose and lactate concentrations) were investigated in controlled laboratory conditions. Aquaria were divided in half with a watertight black acrylic divider sealed with silicone to create two 19 l experimental cells. Each cell measured 25.4 cm (length) × 27.9 cm (width) × 30.5 cm (height). All cells were set up as blinds, with one additional side covered in black plastic sheeting with completely separate water sources, so that fish in one cell could not see or sense fish in any other cell (Schofield, 2004). A standardized volume of clean sand and green ribbon, simulating submerged aquatic vegetation, was uniformly distributed through each cell. The experimental cells were designed to provide equal ratios of cover (ribbon) and open water. Artificial vegetation has been used successfully to mimic natural habitat structure for fishes in laboratory conditions (Keller and Brown, 2008).

Abiotic conditions for the experiment reflected habitat during summer months in lower Corte Madera Creek, within historically occupied habitat of San Francisco Bay Estuary. All aquaria were held in a temperature-controlled circulating seawater table maintained at 21.5 ± 1.3°C in each cell over the duration of each experiment. Replicate cells were within a maximal daily range of 0.5°C of each other. Salinity levels within all aquaria were held at 25.5 ± 0.6 ppt. Air stones kept dissolved oxygen levels of 7.45 ± 0.7 mg O_2_ l^−1^. Internal cell circulation and water quality were maintained for each cell separately with aquarium filters (Whisper PF10; Tetra, Blacksberg, VA, USA) fitted with fine mesh screens to reduce water velocities in each cell. Temperature and oxygen levels of each cell were measured every other day (YSI model 85 meter; YSI Incorporated, Yellow Springs, OH, USA), and water ammonia, nitrate, nitrite and pH of each cell were measured twice a week (Saltwater Master Test Kit; API, Chalfont, PA, USA). Water changes of 5–15% of cell volume were conducted daily to maintain water quality.

To test the effects of food availability on competition between *E. newberryi* and *L. parva* and between *E. newberryi* and *G. aculeatus*, two 28 day experiments were conducted in series, in which two different feeding rations were provided (for details, see next subsection). Each experiment had three fish assemblages for *E. newberryi* to account for both intraspecific competition between individuals of *E. newberryi* and interspecific competition between individuals of different species (two fish species per assemblage). A total of 12 fish were held in each cell under one of three assemblages. Each assemblage had six replicate cells. Cell assemblages included the following: (i) six *E. newberryi* vs. six *E. newberryi*; (ii) six *L. parva* vs. six *E. newberryi*; and (iii) six *G. aculeatus* vs. six *E. newberryi*.

### Feeding regimen

A 3 day feeding trial pilot study was conducted with six *G. aculeatus*, six *L. parva* and three *E. newberryi* to determine the appropriate ration to result in competition for food between fishes in the experimental trials. While variation was observed between species and day, mean daily satiation was found to be ∼10 *Daphnia pulex* per fish (data not shown). For the competition trials, feeding levels were set at 50% (five *D. pulex* per fish; 60 *D. pulex* per cell with 12 fish) and 75% rations (7.5 *D. pulex* per fish; 90 *D. pulex* per cell rounded up as necessary to reach a whole number). Additionally, once a week, 1.5 ml per cell of frozen brine shrimp (*Artemia* spp.; San Francisco Bay Brand, Inc., Newark, CA, USA) was added with the live *D. pulex* fed to each cell. The food source was selected based on the ability to culture and provide a primarily live invertebrate prey base for the experiment and published accounts of each species' diet (Crawford and Balon, 1994; Swenson and McCray, 1996; Spilseth and Simenstad, 2011). In nature, *E. newberryi* consume a broad range of invertebrate prey that changes based on availability but includes primarily crustacean, dipteran larvae, invertebrate eggs and gastropods (Swift *et al*., 1989; Swenson, 1999). Previous laboratory studies by our group and others have used *D. pulex* to feed *E. newberryi* (Swenson, 1999; Chase and Todgham, 2016).

Fish were fed live *D. pulex* cultivated on Phyto-Feast^®^ (Reed Mariculture Inc., Campbell, CA, USA). *Daphnia pulex* were hand counted and separated into two labelled 15 ml conical vials per cell. Feedings occurred once daily, such that the contents of one conical tube were poured randomly around the top of a cell, allowing the *D. pulex* to swim throughout each cell. Approximately 10 min later, after all food had been consumed, the second conical tube with the remaining food was poured into the cell. The staggered feeding approach reduced the volume of food released at a given time during each daily feeding to encourage greater competition for food (Milinski and Parker, 1991).

### Growth measurements

Fish length and weight were measured every 14 days, with measurements on day 1 (when moved to experimental cells), day 15 (experimental midpoint) and day 29 (end of experiment). All fish were netted and temporarily held in an aerated container. Fish were then individually removed for measurement and returned to a separate aerated container to ensure that individuals were counted only once. Weight measurements were taken according to Anderson and Neumann (1996), where live fish were lightly blotted dry and placed in tared 25 ppt seawater-filled weigh boats and weighed to the nearest 0.001 g (Mettler Toledo XS105 dual range microscale; Mettler-Toledo, LLC, Columbus, OH, USA). Standard length measurements were made to the nearest 0.5 mm using dial callipers. As fish were not marked, specific individuals could not be tracked through the experiment.

On day 29, all fish in a cell were captured within a 5 min period and euthanized with a lethal concentration of tricaine methanesulfonate (>250 mg l^−1^; Finquel^®^ MS-222; Argent Chemical Laboratories, Redmond, WA, USA). Fish were measured for length and weight and then flash frozen in liquid nitrogen or on dry ice. Specimens were then stored at −80°C until biochemical analyses.

Length and weight data were used to calculate the relative condition factor for *E. newberryi*. The mean relative condition factor was compared between treatments (Anderson and Neumann, 1996; Froese, 2006). Length and weight data from 118 *E. newberryi* measured at Salmon Creek Lagoon, reported by Chase and Todgham (2016), were used to determine *a* = 0.01647 and *b* = 2.66 values to inform an individual's relative condition factor (*Kn*) using the following equation:

$$Kn=\frac{W}{aL^{b}}$$

where *W* is weight (in grams), *L* is standard length (in centimetres), *b* is the slope of the regression line, and *a* is the intercept value.

### Biochemical analyses of generalized stress response

#### Whole-body homogenization

Homogenization of the entire fish body was performed on 43 *E. newberryi* from the 75% ration (*n*_day 0_ = 6, *n*_day 29_ = 37) and 53 *E. newberryi* from the 50% ration (*n*_day 0_ = 7, *n*_day 29_ = 46). Homogenization procedures followed the protocol used by Hasenbein *et al*. (2013) and included the following steps for each individual sample. Prior to homogenization, samples were weighed while frozen and then quartered over ice. Each sample was then homogenized in 1 ml ice-cold 1× phosphate-buffered saline (PBS buffer: 4.3 mM sodium phosphate, 136.8 mM sodium chloride, 2.7 mM potassium chloride and 1.47 mM potassium phosphate, pH 7.4). Homogenizer blades were washed with 1 ml PBS buffer, which was then combined and vortexed with the sample. The sample was then split in half, with half dedicated to immediate cortisol analyses and half for glucose and lactate analysis. The homogenate required for glucose and lactate analysis was centrifuged for 30 min at 14 500***g*** at 4°C. Supernatant was then extracted to a new centrifuge tube and stored at −80°C until analysis.

#### Cortisol extraction and analysis

Cortisol extraction was performed using methodology adopted for zebrafish, *Danio rerio* (Alsop and Vijayan, 2008; Cachat *et al*., 2010), optimized for juvenile delta smelt, *Hypomesus transpacificus* (Hasenbein *et al*., 2013), and used previously to quantify whole-body cortisol in *E. newberryi*, *L. parva* and *G. aculeatus* (Chase and Todgham, 2016). Tissue homogenate was spiked with 2.0 ml diethyl ether, vortexed and centrifuged for 15 min at 3200*g* at 4°C. Supernatant was extracted without touching the pellet and transferred to a new glass test tube. Preliminary analysis determined that more than 90% of total body cortisol was extracted with a single wash of the tissue homogenate. For the experiment, this process was repeated two more times for maximal extraction of cortisol from the tissue homogenate with diethyl ether (Cachat *et al*., 2010; Hasenbein *et al*., 2013), and the supernatants from the three washes were combined. The pellet was then discarded, and the extracted supernatant in the glass test tube was left overnight in a fume hood to ensure that the diethyl ether fully evaporated following Cachat *et al*. (2010). The following day, samples were resuspended in 1 ml PBS buffer, vortexed for 30 s and stored at 4°C overnight. Cortisol was then measured using an enzyme-linked immunosorbent assay, following the manufacturer's instructions (Neogen Corporation, Lansing, MI, USA). Cortisol was run in duplicate, and mean blank absorbance values were subtracted from each sample. Cortisol concentrations were calculated with a four-parameter sigmoid standard curve. Calculated cortisol concentrations were then corrected for dilution and standardized by sample weight (50% of fish weight used for cortisol) and are presented as nanograms of cortisol per gram of fish wet weight (Supplemental material, Table S1).

#### Glucose and lactate analyses

Frozen tissue homogenate samples were thawed on ice and analysed for glucose and lactate using a Bioanalyzer YSI 2700 (YSI Incorporated, Yellow Springs, OH, USA). Glucose and lactate concentrations were then corrected for dilution and standardized by sample weight (50% of fish weight used for glucose and lactate analyses).

### Statistical analysis

Response variables for *E. newberryi* included survival, length, weight, *Kn*, cortisol, glucose and lactate concentrations. Statistical analyses were conducted in R (version 2.15.0; R Development Core Team 2012). Data were first tested for normality and heteroscedasticity visually by plotting Q-Q, density and residual plots of the linear model to ensure that parametric analysis assumptions were met. Survival (number of alive fish per day compared with the initial 36 fish of that species in a treatment assemblage) for each ration was assessed using a Kaplan–Meier curve with 95% confidence intervals and a χ^2^ analysis, with the null hypothesis that survival rates should remain constant across fish assemblages. Two-way ANOVA (type II) was used to assess the effects of fish assemblage on growth (weight, length and *Kn*) separately within each food ration while accounting for unbalanced fish numbers attributable to mortality. Time (day 1, 15 and 29) and fish assemblage were treated as fixed, discrete effects, with tank replicate as a random effect. Significant *F* values were followed up with Tukey's *post hoc* test on least-squared means to detect group differences (lsmeans v2.20-2; http://CRAN.R-project.org/package=lsmeans). Weight data was logarithmically transformed in the 75% ration to meet the assumption of normally distributed residuals. Repeated measures could not be used with this data set because individual fish were not individually marked and therefore not tracked through the experiment duration.

A two-way ANOVA was used to examine the effects of fish assemblage and feeding ration on physiological indicators of stress (cortisol, glucose and lactate). As the feeding trials were run sequentially, initial median values of each stress indicator from fish sampled on day 0 (before experimental treatment allocation) were subtracted from each fish sampled at the end of the experiment (day 29) for each feeding trial to account for any differences in starting condition, providing net concentrations of cortisol, glucose and lactate. Absolute concentrations of cortisol, glucose and lactate for all groups are provided in the Supplemental material, Table S1. Experimental tank was included as a random effect to account for potential non-independence of fish from the same replicated unit. As all biochemical indicators exhibited heterogeneity in one or both fixed effects, variance was incorporated into the mixed model using the varIdent option in the nlme package. Significant *F* values were followed up with Tukey's *post hoc* test on least-squared means to detect group differences. To assess relationships between the response variables and feeding ration, correlation analysis (Pearson's *r*) was used to measure the strength, direction and significance between *Kn*, cortisol, glucose and lactate in day 29 fish within each ration. The threshold for significance was set at *P* < 0.05. All data are reported as the mean ± SEM unless stated otherwise.

## Results

### Survival

**Figure 1:**
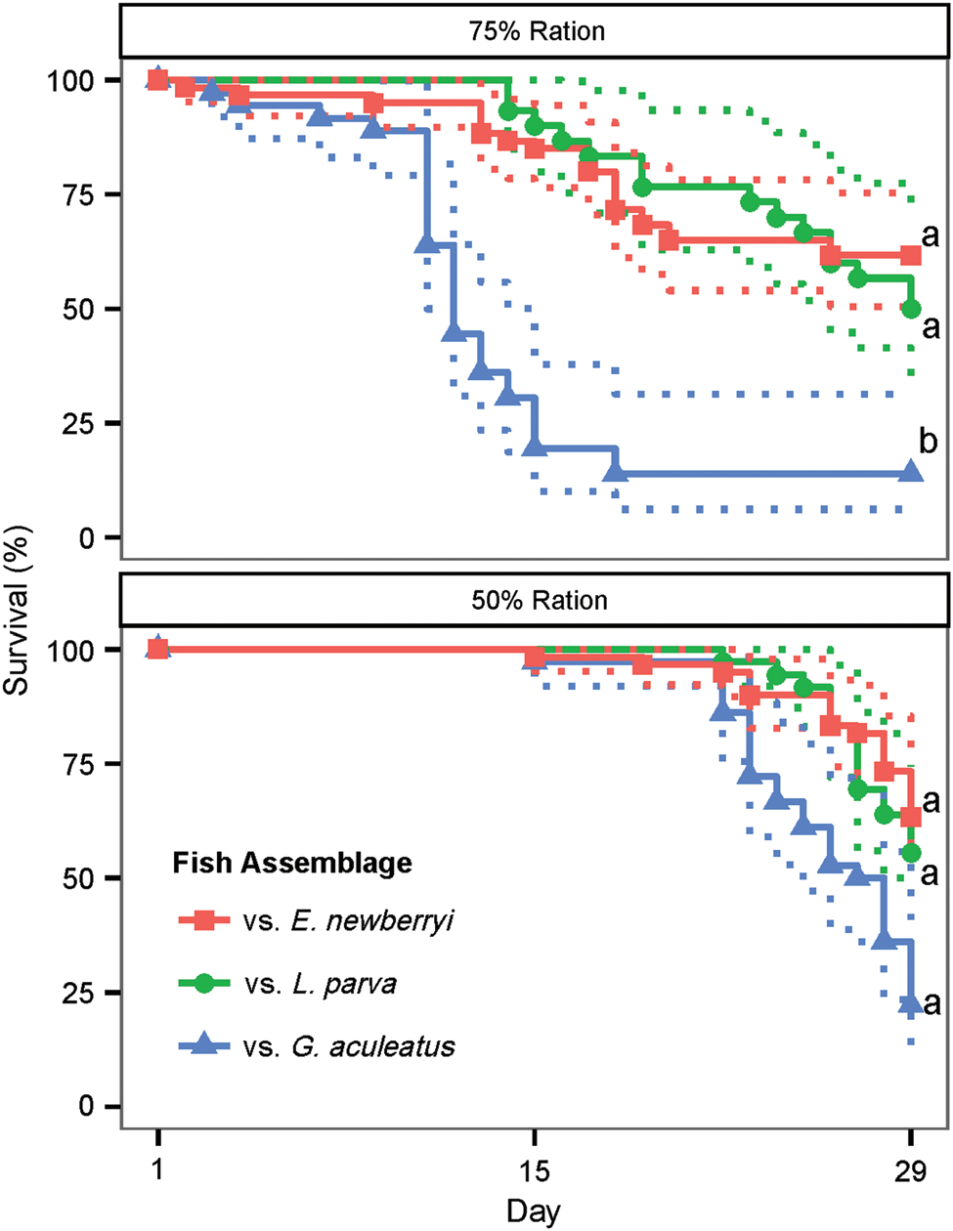
Percentage survival of *Eucyclogobius newberryi* in the 75 and 50% ration trials in the presence of other *E. newberryi* (red squares), *Lucania parva* (green circles) or *Gasterosteus aculeatus* (blue triangles) from day 1 to day 29. Kaplan–Meier curves were plotted with 95% confidence intervals for *n* = 30–60 fish per fish assemblage. Differences in letters represent statistically significant differences (*P* < 0.05) between fish assemblages.

When fed the 75% ration, *E. newberryi* experienced significantly reduced survival in the presence of *G. aculeatus* compared with *E. newberryi* in the presence of either *L. parva* or conspecifics (Fig. 1; Pearson's χ^2^ test, χ^2^ = 9.18, d.f. = 2, *P* = 0.01). Only 13.9% of *E. newberryi* survived to day 29 when held with *G. aculeatus*. Survival to day 29 was highest for *E. newberryi* in the presence of conspecifics, at 61.7%, followed by those held with *L. parva*, at 50.0%.

**Figure 2:**
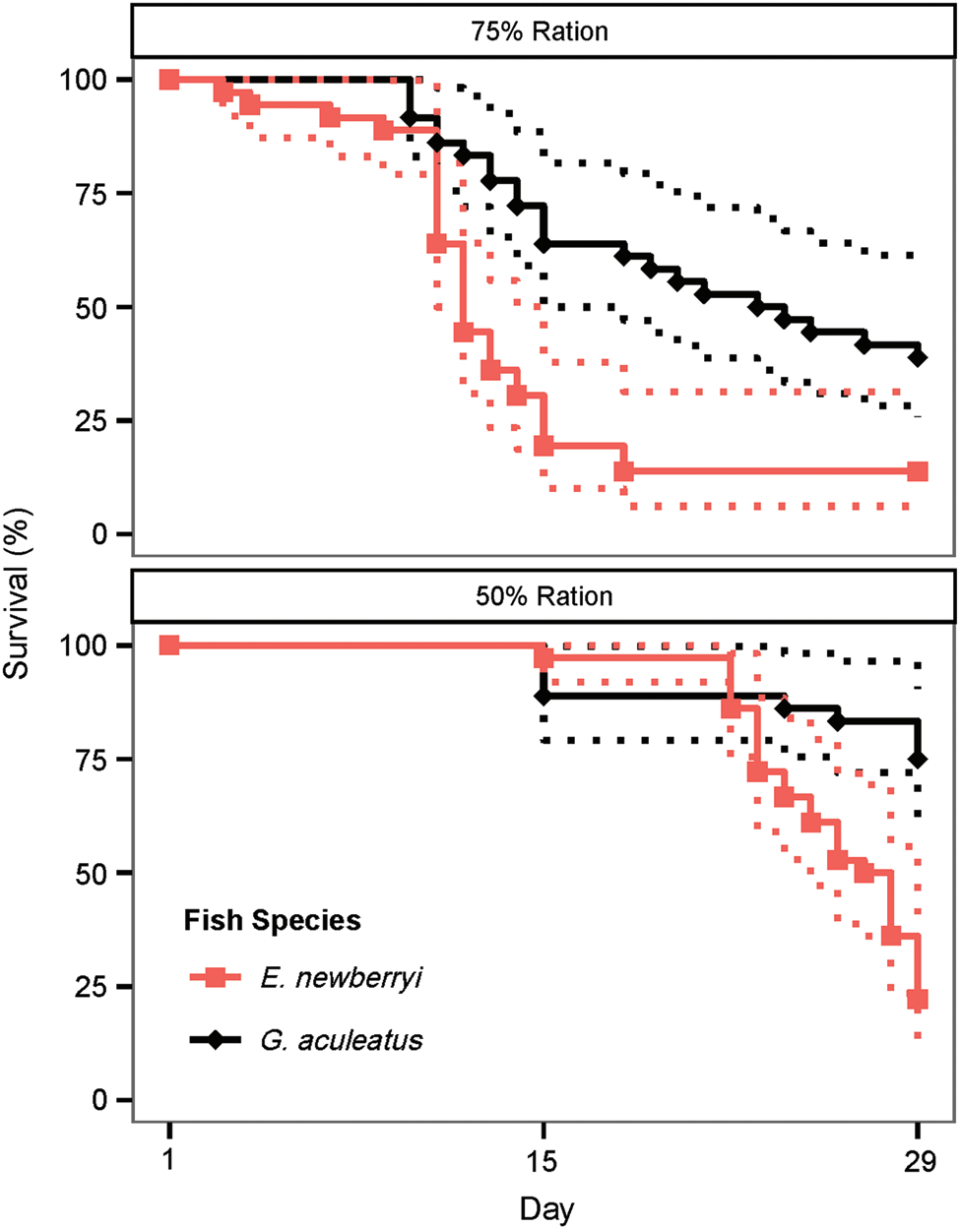
Survival percentage of *E. newberryi* (red squares) and *G. aculeatus* (black diamonds) when held together and fed a 75 or 50% ration from day 1 to day 29. Kaplan–Meier curves were plotted with 95% confidence intervals for *n* = 36 fish per species per ration level.

Survival of *E. newberryi* when fed the 50% ration had a similar trend to the 75% ration; however, mortality began later in the experiment and survival was not quite significantly different between the three fish assemblages at day 29 (Pearson's χ^2^ test, χ^2^ = 5.89, d.f. = 2, *P* = 0.053). *Eucyclogobius newberryi* had the lowest survival at day 29 in the presence of *G. aculeatus*, at 22.2%, compared with 63.3% with conspecifics and 55.5% with *L. parva* (Figs 1 and 2).

### Growth

**Figure 3:**
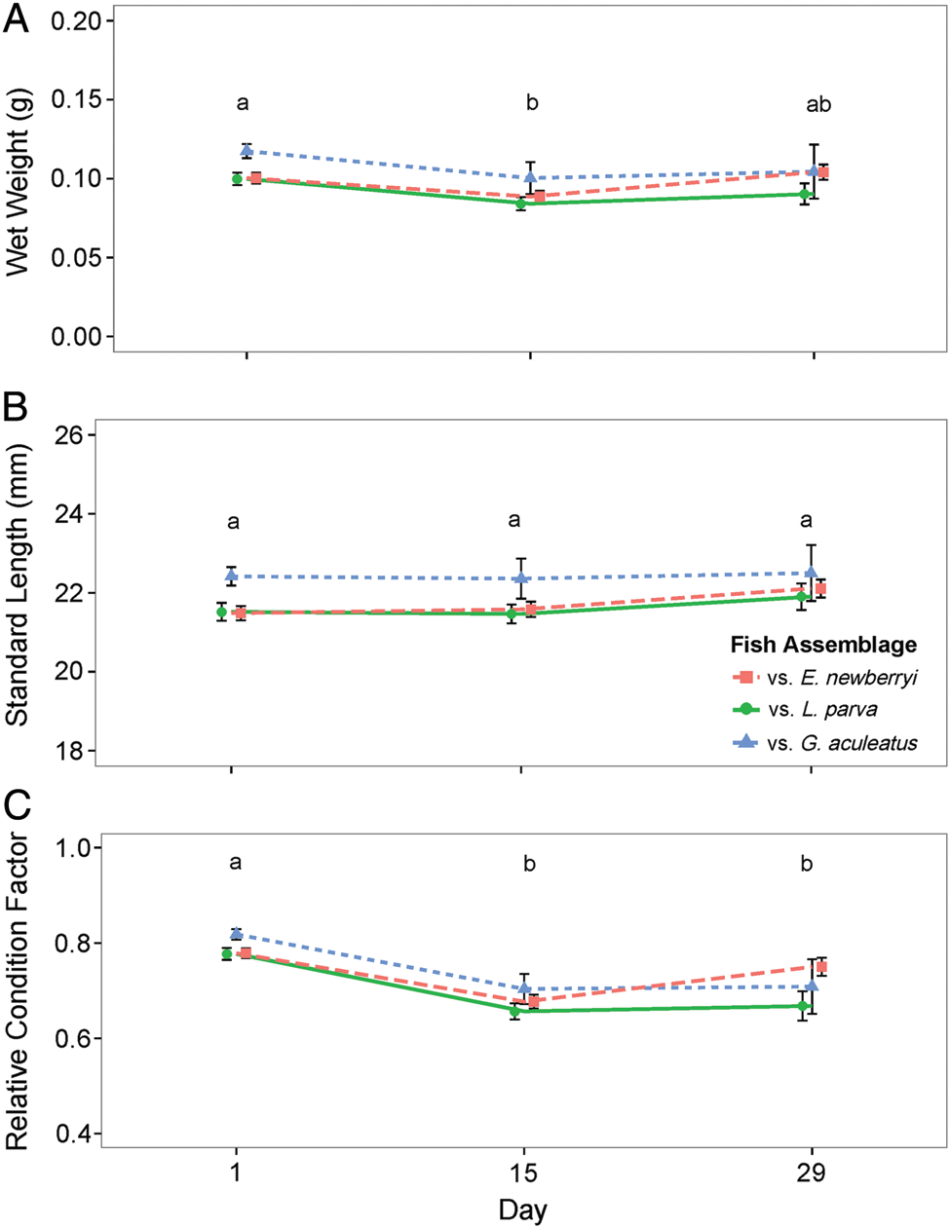
Wet weight (**A**), standard length (**B**) and relative condition factor (**C**) of *E. newberryi* in the 75% ration trial in the presence of other *E. newberryi* (red squares), *L. parva* (green circles) or *G. aculeatus* (blue triangles) on days 1, 15 and 29. Differences in letters represent significant difference (*P* < 0.05) between days. Data represent means ± SEM for *n* = 7–60 fish (variation in sample size attributable to levels of increased mortality during the feeding trial).

When fed the 75% ration, the weight of *E. newberryi* significantly changed over time (Fig. 3A; *F*_2,246_ = 16.93, *P* = 0.0002), but fish assemblage did not affect how weight changed over time (*F*_4,246_=3.10, *P* = 0.54). Fish weight decreased significantly between day 1 and day 15 (0.102 ± 0.003 vs. 0.088 ± 0.004 g), after which it increased to an intermediate value at day 29 (0.095 ± 0.005 g). There was no significant change in fish length over time (Fig. 3B; *F*_2,246_ = 5.20, *P* = 0.074) and no effect of fish assemblage on length over time (*F*_4,246_ = 0.62, *P* = 0.96). Time significantly affected *Kn* (Fig. 3C; *F*_2,246_ = 83.26, *P* < 0.0001), but fish assemblage did not affect how *Kn* changed over time (*F*_4,246_ = 5.81, *P* = 0.21). The condition factor was significantly greater on day 1 (0.79 ± 0.01) compared with day 15 (0.67 ± 0.02) and day 29 (0.70 ± 0.02), accounting for increasing residual variance over time (*L* = 20.28, d.f. = 2, *P* < 0.0001). Although the overall effect of fish assemblage on *Kn* was not significant, by the end of the experiment *E. newberryi* held with conspecifics were on average more similar to initial *Kn* (−4%) compared with the other fish assemblages (with *L. parva* −15%; with *G. aculeatus* −16%).

**Figure 4:**
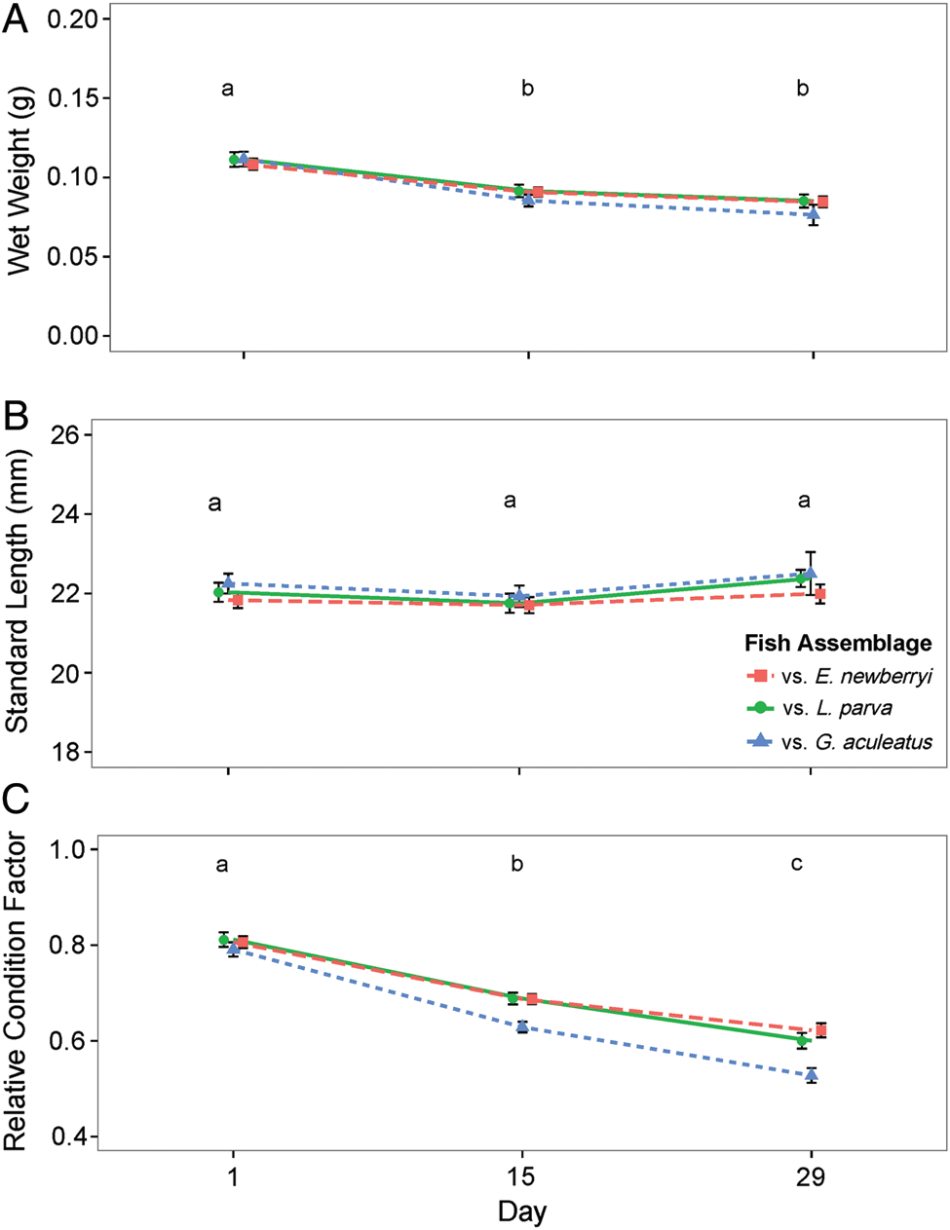
Wet weight (**A**), standard length (**B**) and relative condition factor (**C**) of *E. newberryi* in the 50% ration trial in the presence of other *E. newberryi* (red squares), *L. parva* (green circles) or *G. aculeatus* (blue triangles) on days 1, 15 and 29. Differences in letters represent significant difference (*P* < 0.05) between Days. Data represent means ± SEM for *n* = 20–59 fish (variation in sample size attributable to levels of increased mortality during the feeding trial).

When fed a 50% ration, the weight of *E. newberryi* changed significantly over the course of the experiment (Fig. 4A; *F*_2,327_ = 72.23, *P* = <0.0001), but there was no effect of fish assemblage on how weight changed over time (*F*_4,327_ = 2.45, *P* = 0.65). Fish weight was highest on day 1 (0.110 ± 0.003 g) and significantly less on days 15 (0.089 ± 0.002 g) and 29 (0.081 ± 0.003 g), incorporating decreasing residual variance over time (*L* = 9.39, d.f. = 2, *P* < 0.009). Fish length did not change significantly over time (Fig. 4B; *F*_2,327_ = 3.93, *P* = 0.14), and there was no effect of fish assemblage on fish length over time (*F*_4,327_ = 3.93, *P* = 0.14). The condition factor changed significantly over time (Fig. 4C; *F*_2,327_ = 334.06, *P* = <0.0001) irrespective of fish assemblage (*F*_4,327_ = 7.72, *P* = 0.10). The condition factor was greatest on day 1 (0.80 ± 0.01) and continued to decline significantly over time (day 15 = 0.68 ± 0.01; day 29 = 0.58 ± 0.01). Although we did not detect overall significant effects of fish assemblage on *Kn*, average *Kn* decreased the least from day 1 to day 29 when *E. newberryi* were with conspecifics (−23%) in comparison to being with *L. parva* (−27%) or *G. aculeatus* (−34%).

### Whole-body cortisol concentrations

**Figure 5:**
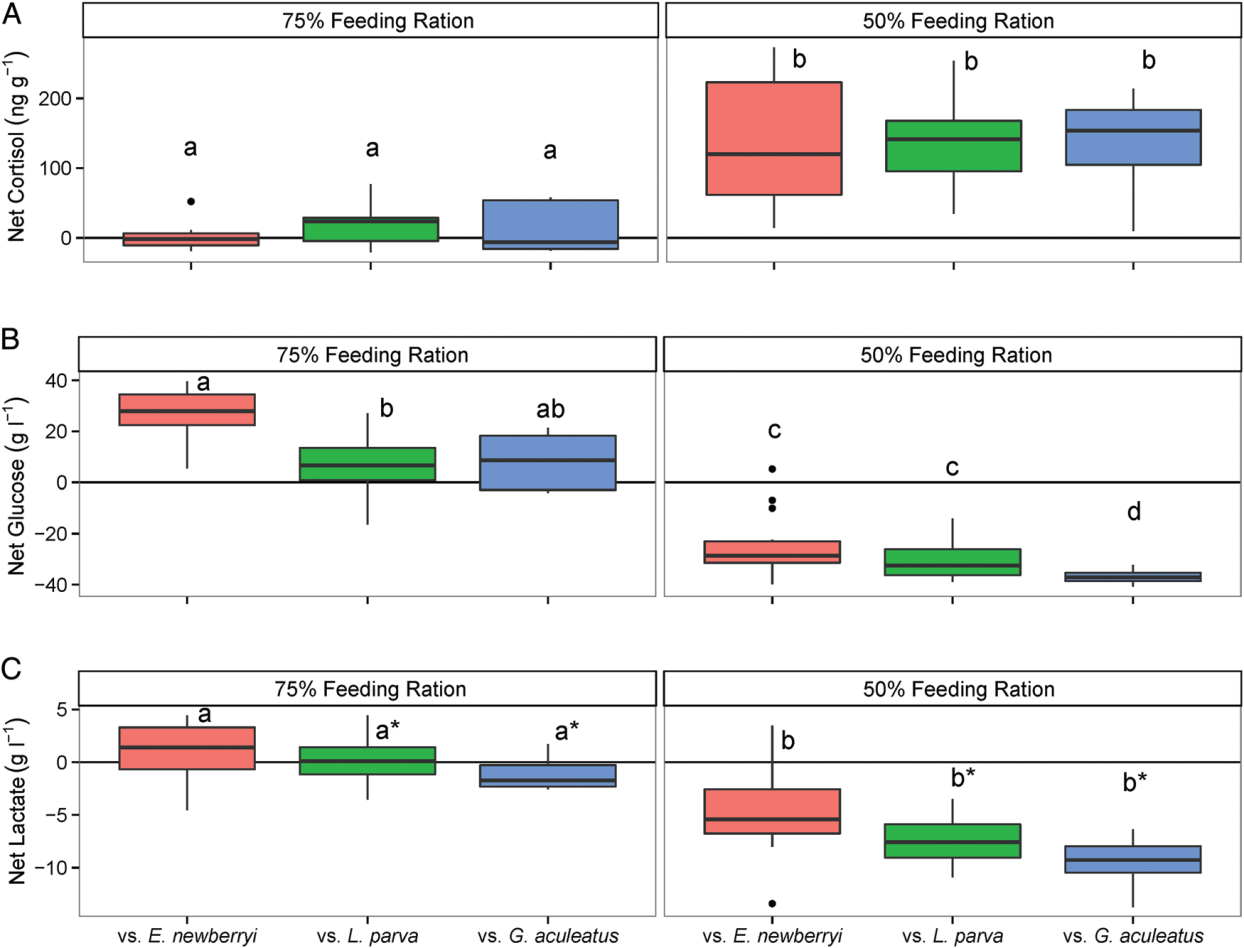
Net cortisol (**A**), glucose (**B**) and lactate concentrations (**C**) of *E. newberryi* measured on day 29 compared with day 0 (after acclimation, before experimentation) in the presence of conspecifics (vs. *E. newberryi*; red), *L. parva* (vs. *L. parva*; green) or *G. aculeatus* (vs. *G. aculeatus*; blue). Left panel reports 75% ration (vs. *E. newberryi n* = 18; vs. *L. parva n* = 14; vs. *G. aculeatus n* = 5). Right panel reports 50% ration (vs. *E. newberryi n* = 18; vs. *L. parva n* = 20; vs. *G. aculeatus n* = 8). For each boxplot, the horizontal line represent the median, the box represents the interquartile range, and the whiskers extend 1.5 times the interquartile range. Points beyond the whiskers are outliers. Differences in lowercase letters represent statistically significant differences (*P* < 0.05) across feeding rations. Asterisks represent concentrations of lactate in *E. newberryi* that are significantly different (*P* < 0.05) between the fish assemblages within a feeding ration.

The feeding ration had a significant effect on net cortisol concentrations (*F*_1,20_ = 144.46, *P* < 0.0001), whereas there was no significant effect of fish assemblage (*F*_2,20_= 3.00, *P* = 0.07) or an interaction between feeding ration and fish assemblage (*F*_2,20_ = 0.48, *P* = 0.63; Fig. 5A). Fish from all assemblages fed a 50% ration exhibited greater net cortisol concentrations [135 ± 12 ng cortisol (g fish)^−1^] compared with fish fed a 75% ration [11 ± 6 ng cortisol (g fish)^−1^]. Feeding ration and fish assemblage had a significant effect on the variability of net cortisol (*L* = 48.98, d.f. = 5, *P* < 0.0001), with all fish assemblages exhibiting a greater range of cortisol values in the 50% ration trial, and *E. newberryi* held with conspecifics exhibiting the greatest overall variation when fed a 50% ration and the least overall variation when fed a 75% ration.

### Whole-body glucose concentrations

Feeding ration interacted with fish assemblage to have a significant effect on net glucose values of *E. newberryi* (*F*_2,20_ = 3.92, *P* = 0.037), with a significant effect of both feeding ration (*F*_1,20_ = 646.29, *P* < 0.0001) and fish assemblage (*F*_2,20_ = 20.19, *P* < 0.0001; Fig. 5B). When fed a 50% ration, all net glucose concentrations were below initial starting values, with *E. newberryi* kept with conspecifics and with *L. parva* exhibiting significantly different net glucose values than when kept with *G. aculeatus*. When fed a 75% ration, all values were significantly different from fish in the 50% ration trial, with the net glucose concentrations of *E. newberryi* with conspecifics being significantly greater than when kept with *L. parva*, and *E. newberryi* kept with *G. aculeatus* overlapping the other two fish assemblages. The best final model included variance by the interaction of feeding ration and fish assemblage (*L* = 17.39, d.f. = 5, *P* = 0.004), as *E. newberryi* kept with *L. parva* and *G. aculeatus* exhibited less variation in the 50% ration compared with the *E. newberryi* kept with conspecifics at the 50% ration and all fish assemblages at the 75% ration.

### Whole-body lactate concentrations

The feeding ration had a significant effect on net lactate concentrations (*F*_1,20_ = 181.92, *P* < 0.0001) as well as fish assemblage (*F*_2,20_ = 9.38, *P* = 0.001), but there was no significant interaction (*F*_2,20_ = 1.58, *P* = 0.23; Fig. 5C). Fish from all assemblages fed the 50% ration exhibited lower net lactate concentrations (−7.2 ± 0.4 g l^−1^) compared with fish fed the 75% ration (0.0 ± 0.5 g l^−1^). *Eucyclogobius newberryi* held with conspecifics had slightly greater lactate values irrespective of feeding ration (−1.8 ± 0.6 g l^−1^) compared with *E. newberryi* kept with *L. parva* (−3.6 ± 0.4 g l^−1^) or *G. aculeatus* (−5.3 ± 0.6 g l^−1^). However, net lactate concentrations were also more variable in *E. newberryi* held with conspecifics compared with the other two fish assemblages (*L* = 8.46, d.f. = 2, *P* = 0.015) across both feeding rations.

### Correlation analysis of response variables

**Figure 6:**
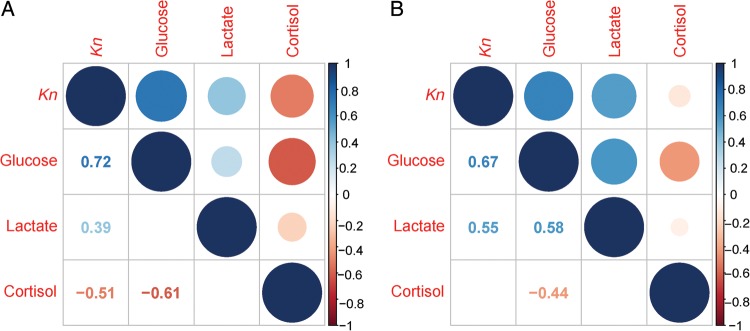
Correlation of response variables [condition factor (*Kn*), glucose, lactate and cortisol] in day 29 fish fed a 75% (**A**) and 50% ration (**B**). The circles in the upper diagonal are proportional to the strength of the correlation (positive values are in blue; negative values are in red), and the numbers in the lower diagonal represent the correlation value (Pearson's *r*) when the significance is *P* < 0.05.

In both feeding rations, there were significant correlations between response variables (Fig. 6). The condition factor in both feeding rations was positively correlated with glucose (75%, *r*^2^ = 0.72, *P* < 0.0001; 50%, *r*^2^ = 0.67, *P* < 0.0001) and lactate (75%, *r*^2^ = 0.39, *P* = 0.016; 50%, *r*^2^ = 0.55, *P* < 0.0001), whereas glucose and cortisol were negatively correlated under both feeding rations (75%, *r*^2^ = −0.61, *P* < 0.0001; 50%, *r*^2^ = −0.44, *P* = 0.003). When *E. newberryi* were held under the 75% ration, there was also a significantly negative correlation between *Kn* and cortisol (*r*^2^ = −0.51, *P* = 0.001) not present in the 50% ration. Under the 50% ration only, glucose and lactate were also positively correlated (*r*^2^ = 0.58, *P* < 0.0001).

## Discussion

Periods of limited food availability can increase competitive interactions between species, potentially resulting in the displacement or removal of an inferior competitor. This dynamic can play an important role in the timing and location of reintroduction efforts, as resident species found in the habitat will interact with the reintroduced species. If reintroduction results in competition among fishes, physiological mechanisms used by an individual to respond to the biotic interactions and maintain homeostasis can be energetically expensive. If the biotic interactions result in chronic stress, the potential for allocation of energy away from growth and reproduction (Sokolova *et al*., 2012) in response to competition could limit the success of a reintroduction. In the present study, we explored the competitive interactions between juvenile *E. newberryi* with a native (*G. aculeatus*) and a non-native species (*L. parva*) at two different ration levels in laboratory conditions. Competition was assessed through survival, growth and physiological indicators of stress (i.e. cortisol, glucose and lactate) of juvenile *E. newberryi* in a food-limited environment, with the objective of gaining a better understanding of species assemblage dynamics for the endangered *E. newberryi*. Overall, our results suggest in controlled laboratory conditions that the availability of food resources affects *E. newberryi* growth and survival during the juvenile phase, with the greatest effects resulting from competition with *G. aculeatus*. Furthermore, when fed a 50% ration, *E. newberryi* experienced chronic stress.

### Survival and growth

*Gasterosteus aculeatus* were able to outcompete *E. newberryi* for food, resulting in reductions in growth (Fig. 4) and increased mortality (Fig. 1) for *E. newberryi*. Limited food resources are known to impair growth and survival because energy metabolism plays a crucial role in the ability of an organism to sustain basal maintenance functions (Chellappa *et al*., 1995; Froese, 2006; Hansen and Closs, 2009; Segers and Taborsky, 2012). As food levels are diminished, the energy required for basal maintenance, stress response mechanisms and development becomes inadequate, and organisms shift energy use to essential maintenance and survival functions (Sokolova *et al*., 2012). As a result, energy allocation for growth, storage and reproduction ceases until more favourable conditions are encountered (Johnsson *et al*., 2005). In the conditions of this experiment, food levels remained constant and insufficient, which probably prevented *E. newberryi* from maintaining energy stores as the experiment progressed and resulted in a decreased investment in growth and no weight gain (75% ration) or a decrease in weight (50% ration).

Competition for food, and not the abiotic conditions of the experiment, are likely to have resulted in the reduced survival levels observed. The salinity and temperature ranges selected for this experiment were designed to simulate summer conditions within the San Francisco Bay Estuary where *E. newberryi* were previously found and where *L. parva* and *G. aculeatus* currently live. In the presence of ample food, five *E. newberryi* were kept in the same salinity and temperature conditions during the experiment and had 100% survival (data not shown). Competition between species has been shown to impact survival of juvenile fish when a better competitor is able to prevent or limit the access of a lesser competitor to a shared food supply (Ward *et al*., 2006; Wuellner *et al*., 2011). Our findings support a competitive interaction between fishes, because *G. aculeatus* survival also started to diminish as *E. newberryi* mortality increased, and the total amount of food entering a cell decreased in proportion to fish density (Fig. 2). This effect might have been amplified with the experimental set-up, because competition intensity can increase when resources and habitat are restricted to small patches (Ward *et al*., 2006; Pess *et al*., 2011), such as you might expect in isolated aquaria. For *E. newberryi* held with *L. parva* and conspecifics, competitive interactions between individuals appeared to be weaker, and as a result, survival in these two fish assemblages were higher and not different from one another. Taken together, the results of the present study suggest that *E. newberryi* were not dominant competitors and were unable to monopolize prey resources when competing with *G. aculeatus*; however, they were not outcompeted by the non-native *L. parva* for prey.

When comparing trials with different rations, survival at the end of each experiment was similar; however, mortality occurred much earlier when fish were fed a 75% ration. Differences in the onset of mortality between feeding trials suggests that greater levels of competition may have occurred when more prey was available. Similar results have been found with other species, where a higher ration level that was still below satiation generated greater competitive interactions and reduced survival for a less dominant species (McGhee and Travis, 2011; Kakareko *et al*., 2013). When fed a 75% ration, fish in the present experiment were observed to be engaging more actively in scramble competition as an attempt to obtain food first (Ward *et al*., 2006). The observed monopolization of prey items by *G. aculeatus* appeared to drive mortality levels much earlier for *E. newberryi*. For *E. newberryi*, the greater expenditure of energy required for chasing prey during scramble competition, with minimal energy replenishment because of unsuccessful prey capture, could have resulted in a greater exhaustion of essential energy required to maintain basal maintenance and survival functions (Johnsson *et al*., 2005). In contrast, when fed a 50% ration, less scramble competition was observed because *E. newberryi* were observed to spend less time and effort actively chasing prey. Low food availability and food deprivation conditions can result in energy-conserving responses in many species of fish (Sogard and Olla, 1996; Paul and Paul, 1998; De Pedro *et al*., 2003; Gingerich *et al*., 2009). Reduced activity during periods of minimal resources can extend survival for several days, providing valuable time for an organism until more favourable environmental conditions are encountered (Sogard and Olla, 1996; Barton, 2002; Sokolova *et al*., 2012). *Eucyclogobius newberryi* appeared able to conserve enough energy during the first 14 days of the experiment on the 50% ration to maintain high levels of survival; however, as food availability did not improve, energy reserves probably began to deplete, and mortality started to occur in the second half of the experiment. Future research is warranted to investigate the impacts of different levels of interspecific competition between *E. newberryi* and *G. aculeatus* on energy reserves, such as glycogen, lipid and protein stores, to gain a better understanding of the differences in mortality observed between the 50 and 75% feed ration trials.

### Generalized stress response to competition for food

Food limitation resulted in chronic stress of *E. newberryi* as evidenced by elevated cortisol concentrations, a primary indicator of the generalized stress response (Barton, 2002). Previous work conducted with *E. newberryi*, in which positive growth was observed in ample food conditions, found basal mean cortisol concentrations of 13–19 ng cortisol (g fish)^−1^ (Chase and Todgham, 2016). The mean cortisol concentrations in the present experiment were approximately double for the 75% ration [23–42 ng cortisol (g fish)^−1^], and almost 10 times higher for the 50% ration [133–140 ng cortisol (g fish)^−1^], suggesting a more severe stress response (Fig. 5). A similar magnitude of cortisol increase has been found in response to stressors in *G. aculeatus* and rainbow trout, *Oncorhynchus mykiss* (Pottinger, 2002; Ellis *et al*., 2004; Bell *et al*., 2007). As part of the physiological response to competition and limited food, glucocorticoid hormones are released, including cortisol, which serves to increase plasma glucose concentrations through gluconeogenesis to fuel increased energy demand (Renaud and Moon, 1980; Leach and Taylor, 1982; Vijayan *et al*., 1991, 1993). With the sustained release of cortisol and the lack of replenishment of energy reserves, the body can undergo changes in performance, including impaired growth, swimming performance and immune function and modified behaviours, including feeding and aggression (Barton, 2002). As cortisol concentrations of *E. newberryi* did not vary between fish assemblages, food limitation was likely to be the dominant stressor rather than species competitive interactions. Food limitation also resulted in reduced growth of *E. newberryi*, regardless of the fish assemblage, providing evidence that the energy obtained in food-limited conditions was not being allocated to growth, and instead, might have been allocated to dealing with the stress of reduced food. As food availability decreased from a 75 to 50% ration, cortisol concentrations were increased and growth was impaired to a greater degree (Figs 3–5).

When fed a 75% ration, *E. newberryi* exhibited a small increase in cortisol that corresponded to a secondary stress response of increased plasma glucose and with minimal changes to lactate concentrations. Plasma glucose concentrations provide an energy substrate needed for the ‘fight or flight’ response (Barton, 2002). As cortisol concentrations were only moderately elevated and plasma glucose concentrations remained high at the end of the experiment, the ability to mobilize glucose during the 28 day 75% ration trial was probably not compromised. In the experiment, glucose and lactate concentrations were positively associated with *E. newberryi* in better condition, and there was a negative correlation between cortisol and fish condition (*Kn*; Fig. 6). Increased cortisol concentrations have been shown to decrease growth rates in feeding fish, resulting from increased protein turnover rates to provide the energy necessary to cope with stress (Barton *et al*., 1987). The decrease in weight observed during the first 14 days of the 75% ration trial may have been the result of lipids and muscle being catabolized to meet the increased energy demand, although energy stores were not measured throughout the experiment. Those fish able to maintain higher glucose and lactate concentrations and low cortisol concentrations were better able to defend their relative condition during decreased food availability.

When fed a 50% ration, cortisol concentrations were elevated but secondary indicators of stress were suppressed, with a significant negative correlation between cortisol and glucose concentrations (Fig. 6). Decreased glucose with elevated cortisol has been well documented in fishes (e.g. Foster and Moon, 1986; Vijayan *et al*., 1991). One explanation is that elevated cortisol concentrations were no longer able to increase glucose for energy metabolism through gluconeogenesis at the end of the experiment owing to the limited energy stores from food deprivation, such as amino acids or lipids (Suarez and Mommsen, 1987; Vijayan *et al*., 1997). Lactate is also known to be an important substrate for increasing glucose concentrations (Renaud and Moon, 1980; Suarez and Mommsen, 1987) and may explain the decreased lactate concentrations, with high cortisol concentrations in the 50% ration trial. However, decreased glucose concentrations, in addition to decreased lactate concentrations, suggest that glucose was either being used for glycolysis or quickly converted to another metabolite. Increased cortisol concentrations have been shown to increase the repletion of liver glycogen from glucose (Mommsen *et al*., 1999), which could therefore be another explanation for the observed negative correlation between cortisol and glucose (Fig. 6). Lastly, *E. newberryi* had elevated cortisol across all fish assemblages; however, there were fish assemblage-specific differences in glucose and lactate concentrations. In fact, fish assemblage also had a significant effect on net glucose and net lactate in *E. newberryi* fed a 75% ration. Differences in secondary indicators of stress between fish assemblages provide evidence that there were likely to be subtle differences in the stress associated with being held with conspecifics vs. other species; particularly, when *E. newberryi* was held with *G. aculeatus*. These subtle effects of fish assemblage on intermediary metabolism may partly explain differences in mortality of *E. newberryi* held with different fishes, although the mechanistic link requires further study. As previously discussed, metabolic suppression has been found to be a coping mechanism for some species of fish when facing food deprivation (Sogard and Olla, 1996; Barton, 2002; Sokolova *et al*., 2012) and would be worth exploring further in future studies of *E. newberryi*.

While differences in food ration resulted in clear differences in whole-body cortisol concentrations, it is possible that some elevation in cortisol measured in this study was a result of acclimation to higher salinity levels. *Eucyclogobius newberryi* can tolerate variable osmotic conditions and has been found in salinities ranging from 0 to 41 ppt (Swift *et al*., 1989; Swenson, 1999). Given that goby are most often found in closed estuarine systems (such as Salmon Creek, the collection location), the summer months would generally be lower salinity, while the lagoon is closed (Spies and Steele, 2016). It is possible that acclimation to higher salinity levels used for the experiment (5 ppt in Salmon Creek vs. 25 ppt in the experiment, more reflective of San Francisco Bay during the summer) resulted in elevated cortisol to make the necessary osmoregulatory adjustments needed to survive in seawater (McCormick, 1995) and, possibly, affected growth and survival. In the present experiment, cortisol concentrations were not assessed in *E. newberryi* acclimated to 25 ppt in food-replete conditions; therefore, we were unable to determine whether any of the increase in cortisol concentrations was independent of competition for food and was a result of salinity acclimation.

### Implications for *E. newberryi* reintroduction efforts

The results of the present study provide novel evidence that food limitation and interspecific competition are important parameters to consider in *E. newberryi* reintroduction efforts. Under reduced ration levels in the laboratory, competition with the native *G. aculeatus* represented a significant risk to *E. newberryi* growth and survival, whereas competition with the non-native *L. parva* was minimal. Although care must be taken when translating results from laboratory experiments directly to wild populations, these types of laboratory studies can be informative and used to interpret, understand, or anticipate the response found in wild fish species assemblages. For wild populations, food can become limited after stochastic events (e.g. lagoon breaching and large precipitation events), across seasons and as an indirect result of climate change (Swenson and McCray, 1996; Spilseth and Simenstad, 2011). In controlled experimental conditions, we found that the native *G. aculeatus* was a dominant competitor for limited food resources and suffered significant mortality only after *E. newberryi* numbers had diminished. *Gasterosteus aculeatus* can be highly aggressive during feeding events, particularly when resources are limited, and have been shown to exhibit pre-emptive exploitive competition with other native species that inhabit estuarine areas by reducing prey availability (Utne Palm and Hart, 2000; Spilseth and Simenstad, 2011). *Gasterosteus aculeatus* is a shoaling species and appears to be capable of outcompeting *E. newberryi* for invertebrate prey (Utne Palm and Hart, 2000; Moyle, 2002). Therefore, it is possible that *G. aculeatus* would be capable of monopolizing low-food conditions in nature, potentially interacting in a negative manner with *E. newberryi* during reintroduction efforts. The dynamic interactions between *G. aculeatus* and *E. newberryi* were some of the most noteworthy results of this study. Future investigations in field conditions where both species coexist but have the opportunity to feed on a more diverse prey base and different feeding niches is warranted to characterize better the degree to which direct competition from *G. aculeatus* may interfere with reintroduction efforts of *E. newberryi*. Furthermore, additional research could investigate whether asymmetrical shifts in species abundance between these two species in nature are a result of competition.

Survival of the non-native *L. parva* was not impacted by limited food availability in our laboratory conditions. Although the sustained reduction in the availability of food had detrimental effects on survival and growth of *E. newberryi*, *L. parva* experienced no mortality under either feeding regimen for the duration of the experiments. Our results suggest that *L. parva* may require less food to persist than *E. newberryi*, which has implications for wild populations. Furthermore, *L. parva* are capable of tolerating a variable range of salinity, temperature and dissolved oxygen levels and are well adapted for estuarine habitats (Dunson *et al*., 1993; Crawford and Balon, 1996; Fuller *et al*., 2007). When factoring in the high reproductive output and short amount of time required to reach sexual maturation, it becomes evident that the threat *L. parva* could pose to *E. newberryi* could be from displacement rather than direct competition (Crawford and Balon, 1994; Moyle, 2002). Further research is also warranted to investigate the potential for *L. parva* to impact re-establishment of *E. newberryi* by mechanisms other than competition and how these species interact in more natural conditions.

## Supplementary material

Supplementary material is available at *Conservation Physiology* online.

## Funding

This work was supported by funds from San Francisco State University and the University of California Agricultural Experiment Station (grant numbers CA-D-ASC-2252-H and CA-D-ASC-2253-RR) to A.E.T.

## Supplementary Material

Supplementary Data
