# Supplementary material for: Survival, growth and stress response of juvenile tidewater goby, Eucyclogobius newberryi, to interspecific competition for food
Source: Conserv Physiol. 2016 Apr 22;4(1):cow013. doi: 10.1093/conphys/cow013 (PMC4845346; doi:10.1093/conphys/cow013)
Supplement: Supplementary Data [file cow013supp.zip › cow013supp.docx]

**SUPPLEMENTAL MATERIAL**

## **Field and laboratory morphometric data comparison**

*Eucyclogobius newberryi* in the current experiment had a lower growth and higher mortality than found with a previous experiment (reported in Chase and Todgham, 2016). To better understand how final fish condition in our experiment compared with fish of similar size in the field, the length and weight from each fish assemblage on Day 29 was compared with a wild population from Salmon Creek Lagoon. This comparison allowed us to assess whether *E. newberryi* at the end of each feeding experiment were closer in weight per length to a wild population or to those that died during the experiment, providing some insight into general condition at the end of experimentation. Weight was analyzed with a one-way ANOVA with competition treatment nested by length for surviving *E. newberryi* from each competition treatment and a wild population from Salmon Creek Lagoon. For the comparison with the Salmon Creek Lagoon population, if significant differences were encountered, a Scheffe’s test was carried out for pairwise comparisons. Included with this analysis was the length and weight measurement for *E. newberryi* that died when fed the 75% and 50% rations.

When fish were fed a 75% ration, there was a significant difference between groups (Nested ANOVA, F_50,125_=9.98, p<0.0001). On Day 29 of the experiment, *E. newberryi* held with conspecifics (Scheffe Test, p<0.05), *Lucania parva* (p<0.05), and *Gasterosteus aculeatus* (p<0.05) had a significantly lower weight to length relationship than *E. newberryi* from the wild Salmon Creek Lagoon population. In general, *E. newberryi* from the conspecific treatment group had a higher weight to length relationship than those held in the presence of *L. parva* or *G. aculeatus*; however, this difference was not statistically significant (p<0.05). In general, larger *E. newberryi* (>23mm SL) within all three fish assemblages were closer in weight to the wild population than they were to the mortality line (Supplemental Figure 1).

When fish were fed the 50% ration there was also a significant difference between groups (Nested ANOVA F_54,125_=8.89, p<0.0001). *E. newberryi* held with conspecifics (Scheffe Test, p<0.05), *L. parva* (p<0.05), and *G. aculeatus* (p<0.05) had a significantly lower weight to length relationship than *E. newberryi* from the wild Salmon Creek Lagoon population (Supplemental Figure 2). Overall, all *E. newberryi* from the 50% ration trial fell much closer to the mortality line, with the weight to length relationship of *E. newberryi* in the presence of *G. aculeatus* statistically insignificant from the weight to length relationship line of the mortality group (p<0.05) (Supplemental Figure 2).

## **Discussion of field and laboratory morphometric data comparison**

While the patterns of growth were largely consistent for *E. newberryi* across the fish assemblages, there were differences in growth for larger fish between the feeding rations. Of the surviving *E. newberryi*, the larger individuals in the 75% ration trial were closer in weight to the wild population at Salmon Creek Lagoon, while smaller fish fell closer to the weight range of the fish that died during the trial. For the length to weight relationship Salmon Creek Lagoon fish data demonstrated a natural growth relationships in the wild and the mortality curve generated from the experimental data demonstrated fish of a condition that corresponds with death. Larger *E. newberryi* were likely more dominant in intraspecific competition, able to monopolize prey resources and increase their weight faster compared to smaller fish (Hansen and Closs, 2009). The closer each fish’s weight was to the mortality line, the more likely the fish could have died in the upcoming days if the experimental duration of the feeding trial was longer. In the 50% ration trial, most surviving fish on Day 29, regardless of size, were closer to the mortality line than the wild population, indicating their continued survival at a sustained 50% ration was unlikely.

**References**

Chase DA, Todgham AE (2016) Effects of species assemblage on the growth and survival of three species of juvenile California estuarine fishes. *Trans Am Fish Soc* 145: 94-107.

Hansen EA, Closs GP (2009) Long-term growth and movement in relation to food supply and social status in a stream fish. *Behav Ecol* 20: 616–623.


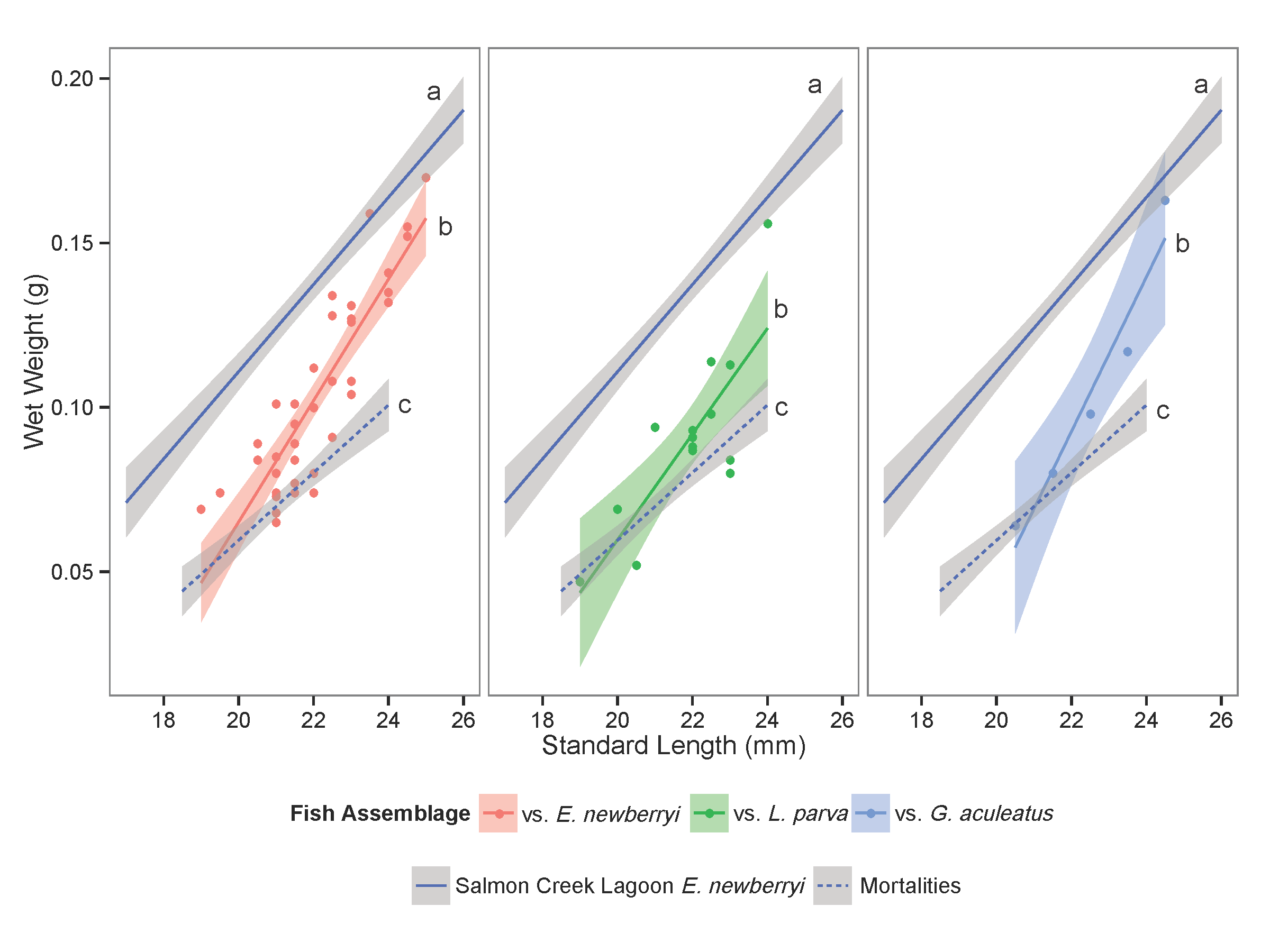


Supplemental Figure 1. Comparison of *Eucyclogobius newberryi* weight to length relationships, plotted as linear trend lines with shaded 95% confident intervals, in the presence of *E. newberryi* (red), *Lucania parva* (green), and *Gasterosteus aculeatus* (blue) on Day 29 of the 75% ration trial. Weight to length relationships of field collected *E. newberryi* from Salmon Creek Lagoon (blue solid line) as well as *E. newberryi* that died during the experiment (mortalities; blue dashed line) are also displayed. Differences in letters represent statistically significant differences in trend lines (p<0.05) between groups.


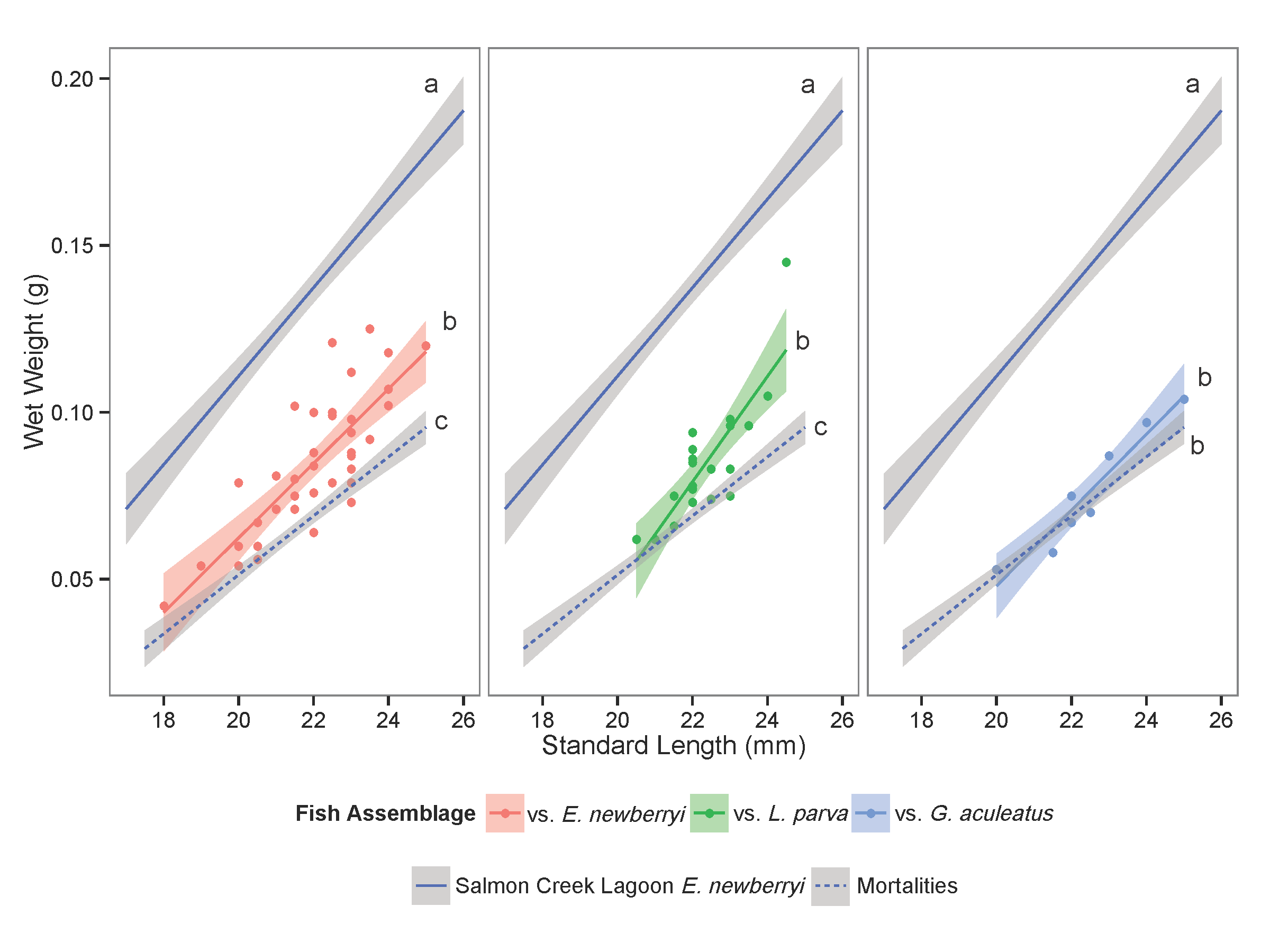


Supplemental Figure 2. Comparison of *Eucyclogobius newberryi* weight to length relationships, plotted as linear trend lines with shaded 95% confident intervals, in the presence of *E. newberryi* (red), *Lucania parva* (green), and *Gasterosteus aculeatus* (blue) on Day 29 of the 50% ration trial. Weight to length relationships of field collected *E. newberryi* from Salmon Creek Lagoon (blue solid line) as well as *E. newberryi* that died during the experiment (mortalities; blue dashed line) are also displayed. Differences in letters represent statistically significant differences in trend lines (p<0.05) between groups.

**Supplemental Table 1**. Cortisol (ng g^-1^), glucose (g l^-1^), and lactate (g l^-1^) levels of *Eucyclogobius newberryi* from the acclimation tank prior to the start of the experiment (Day 0) and measured following 29 days held in the presence of conspecifics (vs. *E. newberryi*), *Lucania parva* (vs. *L. parva*), and *Gasterosteus aculeatus* (vs. *G. aculeatus*) while fed either a 50% or 75% ration. Values are mean ± sem.
